# Marketing and Perceived Value of Differentiated Quality Labels in Extremadura’s Agri-Food Sector

**DOI:** 10.3390/foods14152707

**Published:** 2025-08-01

**Authors:** Alejandro Maya Reyes, Elena Muñoz-Muñoz, Carlos Díaz Caro, Ángel-Sabino Mirón Sanguino

**Affiliations:** 1 Department of Business Management and Sociology, Universidad de Extremadura, Avda. De Elvas s/n, 06006 Badajoz, Spain; amayarey@alumnos.unex.es (A.M.R.); elenamm@unex.es (E.M.-M.); 2 Department of Finance and Accounting, Faculty of Business, Finance and Tourism, Universidad de Extremadura, Avda. de la Universidad, 10071 Cáceres, Spain; asmiron@unex.es

**Keywords:** differentiated quality labels, consumer perception, agri-food marketing, rural development, protected designation of origin, protected geographical indication, willingness to pay

## Abstract

The present study focuses on the attractiveness and perceived value of differentiated quality labels, such as the Protected Designation of Origin (PDO) and Protected Geographical Indication (PGI), for agri-food products from Extremadura (Spain). In doing so, it addresses a gap in the scientific literature concerning consumer behavior toward products bearing these certifications. The results show that awareness of these quality schemes is significantly higher among middle-aged and older individuals, underscoring the need for more modern and targeted communication strategies. The findings highlight the strategic role of agri-food marketing in promoting certified products and emphasize the importance of bridging the generational gap in consumer education. Overall, differentiated quality schemes are perceived as strategic tools to enhance the competitiveness of local products, strengthen cultural identity, and foster sustainable rural economies. Furthermore, this study identifies a negative relationship between the consumption of certified products and the awareness of certification and a positive relationship with the willingness to pay a premium. Consumers with greater awareness tend consume these products less, although they are more willing to pay higher prices for items bearing quality labels.

## 1. Introduction

Differentiated quality schemes such as Protected Designation of Origin (PDO), Protected Geographical Indication (PGI), and Traditional Specialty Guaranteed (TSG) play a central role in enhancing the value of agri-food products, particularly in regions where production is closely linked to local territory and tradition. Regulated by the European Union [1], these schemes ensure that products meet quality standards that go beyond conventional norms and are protected as forms of intellectual property.

PDO certification guarantees that all stages of production take place within the defined geographical area and that the quality of the product is intrinsically tied to that region. In contrast, PGI requires that at least one stage of production occurs in the specified area and that the product’s quality, reputation, or other specific characteristics are directly linked to its geographical origin. Finally, TSG is not associated with a specific geographical location but instead recognizes products that are made using traditional ingredients, composition, or production methods.

The key regulatory and territorial differences among these labels are summarized in Table 1. These labels not only support fair labor conditions and fair wages for producers but also guarantee product authenticity for consumers. In doing so, they support rural development, sustainability, and the preservation of traditional, cultural, and gastronomic heritage.

As noted, a key feature of these quality schemes is their territorial dimension: while TSG emphasizes traditional production techniques, PDO and PGI stress the connection between a product and its geographical environment. In this context, quality is understood not only as a physical or functional attribute but also as a symbolic and cultural element that reinforces regional identity [2].

Extremadura is a notable Spanish region with abundant agri-food resources, where the adoption of these certification schemes has had a significant impact on rural development, gastronomic tourism, sustainability, and economic competitiveness. Extremadura has 13 agri-food quality seals: 10 Protected Designations of Origin (PDOs) and 3 Protected Geographical Indications (PGIs). Although they have great prestige, their economic impact on the regional Gross Domestic Product (GDP) is relatively small.

According to data published by the newspaper *El Periódico de Extremadura* in May 2022, in 2020, the market for PDO and PGI products from this region was worth EUR 74.5 million. That year, Extremadura’s GDP was around EUR 20,000 million, so certified products accounted for about 0.37% of regional GDP. Hence, this work focuses on analyzing the demand for products that have this differentiated quality seal which, in agri-food products, is a key element for rural development and tourism promotion in regions such as Extremadura. The uniqueness of its products, certified by quality figures such as the Protected Designation of Origin (PDO) or the Protected Geographical Indication (PGI), not only guarantees their authenticity and excellence but also contributes to local socio-economic development and the strengthening of territorial identity. The latter is defined by [2].

At the international level, in markets such as the United States or Germany, environmental labels (eco-labels) are gaining importance as a key factor in purchase decisions. Models such as the wine routes in Argentina or the cheese routes in Switzerland have been successful in attracting sustainable tourism and valuing local products.

To explore consumers’ perception and the attractiveness of these differentiated quality labels—whose characteristics are described in the following section—a survey was conducted among Spanish consumers. The methodological design of the survey is detailed in the third section of this paper. The survey gathered information on consumer knowledge, preferences, and attitudes toward certified agri-food products, and the statistical analysis is presented in the Results section. Finally, this paper concludes with a summary of findings to provide a comprehensive view of the role of quality labels in shaping consumer behavior and promoting sustainable rural development.

## 2. Theoretical Framework

From an economic perspective, these certifications serve as mechanisms for generating added value. They enable producers to obtain premium prices while fostering consumer loyalty through guarantees of authenticity, origin, and quality. PDO and PGI certifications contribute to fairer and more sustainable local value chains and act as differentiation tools in an increasingly competitive global market. In particular, agri-food cooperatives play a key role in strengthening rural networks through collaborative production and marketing models [3].

Previous works have analyzed the adoption of the PDO label from different perspectives. From the point of view of agri-food companies, Bouamra-Mechemache and Chaaban [4] found that PDO producers benefit from a premium price that compensates for higher production costs. Kos Skubic et al. [5] analyzed the importance of PDO as a selection criterion when purchasing traditional food products, as well as consumer awareness and determining factors for buying products with PDO labels. Their findings revealed a direct relationship between the belief that PDO indicates higher quality and the use of such labels. Velčovská and Sadílek [6], on the other hand, analyzed the EU quality labeling system.

However, the appeal of these products goes well beyond economic factors. Gastronomy tourism has become a strategic sector in many European regions. In Extremadura, products such as *Torta del Casar* (cheeses), *Jamón Ibérico de Dehesa de Extremadura* (hams), and *Ribera del Guadiana* (wines) contribute to a tourism offering based on authenticity, experience, and territorial connection. In this regard, [7] focused on oleotourism, analyzing three olive oil touristic routes linked to PDOs in Córdoba, namely Baena, Montoro-Adamuz, and Priego de Córdoba, identifying oleotourism as an opportunity to promote rural development. The blending of gastronomy and tourism not only stimulates consumption but also valorizes traditional knowledge, natural landscapes, and cultural heritage, creating a cycle of local development.

As for consumers’ perception, [8] found that price and origin labeling (PDO) are the most influential attributes in consumer preferences. They identified four consumer segments, two of which prioritized origin labels. Ref. [9] examined the relationship between the perceived quality of a PDO-labeled food product—such as ham—and consumer satisfaction and loyalty. Their results showed that perceived quality has a positive influence on both satisfaction and loyalty. The same authors also analyzed [10] the influence of intrinsic and extrinsic perceived quality on consumer satisfaction, trust, and engagement in the case of PDO Somontano wine. They concluded that satisfaction and trust are the main drivers of consumer commitment to PDO, with satisfaction determined by intrinsic quality attributes and trust influenced by extrinsic ones. Finally, they studied [11] the relationships between satisfaction, loyalty, and purchase intention among Spanish consumers. Their research on *Aceite de Oliva del Bajo Aragón* (oil) with PDO found that greater satisfaction leads to higher loyalty and purchase intention. They also emphasized the importance of consumers’ perceptions regarding the association between traditional food products and their place of origin, territory, climate, and local expertise.

To assess the economic and informational effectiveness of geographical indications, Desquilbet and Monier-Dilhan [12] developed a model to evaluate whether PDOs behave as reliable quality signals for consumers. Their findings suggest that the value of these labels depends not only on their ability to certify geographic origin but also on the existence of minimum quality standards. This underscores the relevance of empirical studies—such as the present one—that examine how consumers perceive and respond to quality certifications. By comparing consumer expectations with the predictions of signaling theory and market structure, our results contribute to the ongoing debate on the effectiveness and credibility of geographical labels.

Moreover, consumers’ marginal willingness to pay for products bearing geographical indications shows that while these labels are generally considered quality signals, there is substantial heterogeneity in how consumers value individual PDO and PGI products [13]. This highlights the importance of investigating consumer perceptions at the product level and within specific regional contexts. Our study aligns with this approach by examining consumer attitudes toward certified agri-food products from Extremadura, thereby producing empirical evidence for the ongoing discussion on the credibility and informational value of geographical labels in shaping consumption decisions. Similarly, ref. [14] showed that although geographic origin influences consumer evaluations, the heterogeneity in preferences observed through the mixed logit model suggests the need to explore less conventional socioeconomic variables, such as place of residence, to better capture variation in consumer tastes.

Regarding the alignment with the Sustainable Development Goals (SDGs), particularly SDG 12 (responsible consumption and production) and SDG 8 (decent work and economic growth), these quality schemes promote a more environmentally friendly and socially inclusive agri-food model [15]. In addition, efforts are underway to transition toward circular and regenerative production models. Thus, projects such as *SustaiNext*, which involves the PDO *Torta del Casar*, exemplify how agricultural waste can be reused within sustainable value chains, generating both environmental and social benefits [16,17].

However, the sustainability impact of the production schemes varies depending on the design of the product specifications, producers’ environmental awareness, institutional governance, and the degree of territorial linkage [18]. Nonetheless, from the consumer perspective, sustainability is still not perceived as a strong purchasing incentive due to the limited visibility of these attributes on labels. This points to the need for more effective communication strategies to enhance the credibility and informational value of quality labels in the context of responsible consumption [19].

On a separate note, the successful adoption and management of PDO and PGI schemes require not only financial resources but also substantial investment in social capital, governance structures, and local cooperation networks. The establishment of associations, regulatory councils, and consortia entails complex organizational processes that rely on mutual trust, institutional coordination, and collective action capacity [20]. In this context, the integration of Environmental, Social, and Governance (ESG) criteria into investment decisions provides a strategic pathway for channeling sustainable financing into these quality schemes [21,22,23]. This strengthens not only their market positioning but also their long-term viability.

Beyond economic and environmental dimensions, these schemes also imply deep cultural significance. They foster social cohesion and a sense of belonging in rural areas while preserving traditional techniques and endangered local varieties. Quality thus becomes a strategic pillar of territorial competitiveness: not merely a commercial attribute, but a cornerstone of sustainability and cultural identity [24].

Nevertheless, maximizing the potential of these schemes requires addressing several challenges: limited visibility among younger audiences, the lack of digital communication strategies, and the perception of sustainability as a secondary factor in consumer decision-making [19]. In this regard, agri-food marketing is essential. Strategies that incorporate storytelling, emotional branding, social media campaigns, and smart labeling can enhance awareness, perception, and willingness to pay, especially among younger generations.

Extremadura represents a paradigmatic case: a Spanish region with significant agri-food and tourism potential, rich in high-quality products and provided with a strong identity, poised to strengthen its market position through PDO and PGI schemes. Achieving this requires an integrated strategy that combines innovation, sustainability, communication, and territorial governance. Only then can these quality labels become true drivers of rural transformation and enablers of a fair and competitive transition.

### Hypothesis Formulation

Following [25], this study aims to assess the relevance of quality certifications in consumers’ decision-making, with a specific focus on Spain and the regional context of Extremadura. The goal is to extrapolate the findings to broader dimensions of sustainability, marketing, and social implications associated with sociodemographic factors.

To support the analysis and collect the necessary data, a population-based survey was conducted. The questionnaire was developed and administered using Google Forms. For analytical purposes, the survey was structured into four key sections, enabling a comprehensive exploration of the phenomenon, grounded in the previous literature. Prior to the first section, a filter question was included to segment respondents and ensure proper sample characterization by directly asking whether they were familiar with PDO and PGI certifications.

The first section of the questionnaire focused on the impact of certifications on purchasing decisions. This leads to the first research hypothesis, following the approach taken by Velčovská and Del Chiappa.

**H1:**  *There is a relationship between consumers’ awareness of food quality certifications and their consumption of certified food products.*

Previous studies have shown that quality labels such as PDO and PGI are perceived by consumers as indicators of authenticity and quality [25,26,27,28,29]. These certifications have been found to positively influence trust and product perception, which justifies the formulation of this first hypothesis. Including questions about purchase frequency and the attributes influencing consumer decisions allowed for a deeper understanding of consumer patterns and the relevance attributed to these certifications in the purchasing process.

Ref. [29] also showed that knowledge of these certifications influences both consumer trust and product perception, potentially increasing purchase frequency and willingness to pay a higher price. This leads to the second hypothesis of this study:

**H2:**  *Willingness to pay a higher price for quality-certified products is directly related to awareness of these certifications and results in higher consumption.*

This hypothesis is also supported by previous studies [26,30,31,32]. However, other studies [32,33,34] have offered contrasting evidence. While PDO/PGI labels appear important, there is also evidence [34] that only a small portion of consumers were able to correctly associate the labels with the characteristics they are meant to represent, including organic or geographical production.

In parallel, research has shown that consumers positively value the environmental impact of certified products and consider it important that these certifications ensure environmental respect [35]. Furthermore, a connection has been identified between certified products and rural tourism, as their commercialization may encourage visits to small towns, thereby reinforcing local economies and cultural identity [36]. This perspective links certifications to rural development and their potential to generate both economic and sociocultural benefits in Extremadura. The question “Have you ever visited a municipality in Extremadura motivated by a PDO or PGI product?” was included to assess the influence of certified products on tourism and regional mobility. This gives rise to the third and fourth research hypotheses:

**H3:**  *Consumption of certified products is positively related to their sustainable production.*

**H4:**  *Consumption of certified products is positively related to rural development.*

Finally, the questionnaire included classification questions to segment the sample and analyze potential differences in perceptions and behavior based on sociodemographic variables. Previous studies have indicated that factors such as age, gender, and education level influence the awareness and evaluation of quality certifications [25]. Likewise, place of residence was deemed relevant, as living in Extremadura or in a rural area may be associated with greater familiarity and access to certified products [35]. These questions allowed for better contextualization of the results and the identification of differentiated trends and patterns among consumer groups. This motivated the formulation of the final three hypotheses:

**H5:**  *Consumption of certified products is related to consumer age and associated with awareness of quality certifications.*

**H6:**  *Consumption of certified products is related to consumer gender and associated with awareness of quality certifications.*

**H7:**  *Consumption of certified products is related to consumers’ education level and associated with awareness of quality certifications.*

In summary, the questionnaire was developed based on a thorough review of the scientific literature, which supports the relevance of quality certifications in purchasing decisions, consumer perceptions, and their impact on rural development. The combination of questions addressing awareness, purchasing habits, sustainability, tourism, and sociodemographic variables provided valuable insights for understanding this phenomenon in the region of Extremadura.

## 3. Materials and Methods

### 3.1. Sample Description

The reliability and internal consistency of the questionnaire, as well as the relationships among the questions, are high, as indicated by the Cronbach’s alpha value, presented in Table 2.

The analysis of the survey data was conducted following the methodological guidelines proposed elsewhere [37]. A total of 306 responses were obtained after the questionnaire was closed, and the data were exported to a Google Sheets spreadsheet for structured organization and subsequent processing in Microsoft Excel.

In Excel, a codebook was created, assigning a specific code to each question and a numerical value to each possible answer, following the coding protocols required for accurate interpretation by Stata Version 14. This allowed the proposed regression models to be implemented in order to test the research hypotheses.

The main characteristics of the sample are presented in Table 3, which summarizes the sociodemographic profile of the respondents. Statistically, the dominant profile among the 306 respondents is that of a woman (67.3%), aged between 46 and 60 years (28.1%), with a university degree (40.8%), residing in Extremadura (89.5%), living in a rural area (53.3%), reporting a monthly income between EUR 1200 and EUR 2800 (43.1%), and belonging to a household of four people (29.4%).

**Table 3:** Sociodemographic characteristics of the sample (%).

**Age**	**%**	**Education Level**	**%**
Under 18 years	1.3	Primary Education	8.2
18–25 years	24.5	Secondary Education	10.8
26–35 years	14.1	Vocational Training—High School	25.2
36–45 years	17.6	University Degree	40.8
46–60 years	28.1	Postgraduate Studies	15.0
Over 60 years	14.4		
**Gender**	**%**	**Residence in Extremadura**	**%**
Male	32.7	Yes	89.5
Female	67.3	No	10.5
		Rural Area	53.3
		Urban Area	46.7
**Household Size**	**%**	**Monthly Income**	**%**
One person	6.9	Less than €1200	6.5
Two people	27.8	Between €1200 and €2800	43.1
Three people	28.8	Between €2800 and €3500	15.7
Four people	29.4	More than €3500	16.7
Five or more people	7.2	Prefer not to answer	18.0

Source: Authors’ own elaboration.

### 3.2. Statistical Analysis

To analyze the factors that affect the consumption of products with a designation of origin and quality seal, multiple linear regression was carried out using the Stata v.16 program. The dataset, described in the previous section, was composed of 306 observations, and the dependent variable was consumption. Seven independent variables were included, also described above and shown in Table 4 and Table 5.

The model was estimated using ordinary least squares (OLS) and showed a high explanatory capacity, with an R-squared of 0.9838, which indicates that approximately 98.38% of the variability in consumption is explained by the variables included. The global model is statistically significant (F = 2579.56; *p* < 0.001).

The analysis was complemented with 95% confidence intervals, and the individual significance of each coefficient was evaluated using *t*-tests. The outputs of the model were obtained directly from Stata, guaranteeing accuracy in calculations and statistical reliability. The following subsections explain the results obtained in the model.

## 4. Results

### 4.1. Descriptive Analysis

This section presents a descriptive analysis of the survey results. A large proportion of respondents reported being familiar with differentiated quality certifications—specifically, 75.5% stated that they are aware of them, indicating broad familiarity with such designations, suggesting that these certifications have a high level of incidence and recognition among the population. Moreover, 86.6% of respondents stated they are willing to pay a higher price for a product bearing a differentiated quality certification. This high percentage reveals a positive attitude toward such products and suggests that, for most people, the added value represented by the certification justifies paying a higher price.

A categorical 93.9% responded affirmatively when asked whether they believe that certified products (PDO, PGI, TSG) foster sustainable farming and livestock practices. This response reflects a positive perception and broad recognition of the impact that these certifications can have in promoting more environmentally responsible production methods.

In addition, 76.2% of respondents have indeed visited a locality for this reason. This finding highlights the strong link between differentiated quality certifications and gastronomic tourism, suggesting that such products serve not only as indicators of quality and origin, but also as catalysts for regional economic activity. The data underscore the potential of certified agri-food products to attract visitors, promote local heritage, and contribute to the sustainable development of rural areas through tourism.

The respondents were also asked about the perceived contributions of these certified products to rural development in Extremadura, either creating local employment, preventing rural depopulation, increasing gastronomic tourism, or supporting small-scale producers. The main factor identified as contributing to rural development is the increase in gastronomic tourism, mentioned by 35.1% of respondents, reflecting the growing appreciation of culinary heritage as an economic driver in rural areas. In second place, 28.1% considered that rural development is promoted by supporting small-scale producers, highlighting the importance of strengthening rural economies. Additionally, 22.9% pointed to the creation of local employment as a significant contribution. To a lesser extent, 12.6% perceived that these actions help prevent rural depopulation—an especially critical issue in the region. Finally, only 1.3% stated that they do not believe such initiatives have a significant impact, suggesting broad social acceptance of their value.

### 4.2. Econometric Estimation

Based on the review of the previous literature and the hypotheses proposed in this study, the econometric model described by Equation (1) is estimated in order to present the relationships identified in this work:

(1) Consumption_cp = β₀ β₁^knowledge^ + β₂^willingness_to_pay^ + β₃^sustainability^ + β₄^rural_development^ + β₅^age^ + β₆^gender^ + β₇^education^

In this model, the main variable is the consumption of certified products (Consumption_cp), which is described by the following variables: knowledge of certification (β₁^knowledge^), addressed in H1; willingness to pay for certified products (β₂^willingness_to_pay)^, addressed in H2; the role of certified products in promoting sustainable production (β₃^sustainability^), corresponding to H3; and the contribution of consuming these certified products to rural development (β₄^rural_development^), as per H4. Additionally, the model includes age, gender, and education level (β₅^age^ + β₆^gender^ + β₇^education^, respectively), which are taken into account in H5, H6, and H7, respectively. Table 4 provides descriptive statistics for the variables used in the regression analysis, while Table 5 reports the results of the econometric model described above.

### 4.3. Discussion

The econometric model shows good fit and identifies as statistically significant, at the 5% significance level, two variables associated with the consumption of certified products: knowledge of the certification and willingness to pay more for such products.

The regression shows consistent values, F(7, 298) = 2579.56, with *p* < 0.0001, meaning the general model is very significant. R-squared = 0.9838 indicates that the model explains 98.38% of the variability in the variable “Consumption”. In the same way, the root mean square error (Root MSE) = 0.056 indicates that the average error is low, which indicates good accuracy of the model.

The regression analysis also shows a negative and highly significant relationship between the level of knowledge about certifications and the variable “Knowledge” (coef. = −0.998; *p* < 0.001). This result indicates that, contrary to expectations, greater knowledge about certifications is associated with a lower probability of consuming certified products. Various studies support this trend: research in the Spanish market indicates that more informed consumers can develop a more critical attitude towards certifications, detecting inconsistencies or practices of greenwashing [38]. In addition, it has been observed that label saturation, lack of transparency, or the perception of high prices can generate mistrust and decrease interest in certified products [39]. This phenomenon could reflect a growing preference for more direct consumption practices, such as dealing with local producers or rejecting institutional intermediaries, as argued in [40,41]. At the international level, Luo et al. (2018) [42] propose that knowledge can have nonlinear effects on innovation and behavior, describing an inverse S-shaped curve where excess knowledge can lead to saturation, skepticism, and less willingness to adopt certified products.

The remaining variables are not statistically significant in explaining variation in the dependent variable. However, since the estimated β coefficients for all variables are positive, it can be concluded that they exert a positive effect on the consumption of products with PDO and PGI labels. In particular, greater awareness of certification is associated with increased consumption, a result that aligns with previous works [5,25,26,27,28]. Regarding the variable willingness to pay, it also proves significant and is positively associated with the consumption of products certified under the PDO and PGI quality schemes—that is, there is a willingness to pay a higher price for products endorsed by such certifications, in agreement with previous studies [25,26,30,31,32].

As previously noted in the discussion of the hypotheses, some studies report contrasting findings [33,34,43]. Although PDO/PGI labels appeared to be relevant, only a small proportion of consumers were able to correctly associate the characteristics of PDO/PGI or organic farming with their respective labels [34]. As for the remaining variables, they are not statistically significant, but it is worth noting that most are negatively associated with consumption, except for education level, which shows a positive relationship.

The results of this study reveal a high incidence and social recognition of differentiated quality certifications in Extremadura. Approximately 75.5% of those surveyed are aware of PDO and PGI figures, in agreement with previous studies that highlight these seals as indicators of authenticity and origin [25]. This level of knowledge reinforces the perception of excellence of the certified product and its territorial link as an element of symbolic guarantee. The added value does not lie only in physical attributes, but in traditional practices, traceability, and respect for the environment. This justifies why 86.6% of participants are willing to pay a higher price for certified products, supporting research such as that of [26], which highlights the willingness to pay as an expression of the value received.

Regarding rural development, gastronomic tourism is positioned as the main driver (35.1% of the sample), since it links certified products with cultural experiences that promote the local economy and strengthen regional identity [7].

Certified products are also valued for their contribution to sustainability: 93.9% of those surveyed responded that they favor environmentally friendly practices. Although this attribute is not yet decisive in purchasing, it represents an opportunity to improve the visibility of sustainable practices in labeling, aligning with initiatives such as SustaiNext [16,17], which integrates circular models in PDO chains such as the Torta del Casar.

Finally, relevant sociodemographic nuances are observed. Age and educational level have an impact on the perception and willingness to pay, with older consumers with university degrees assigning greater value to certification [31]. Although gender is not significant in the model, the female predominance (67.3%) in the sample points to a greater involvement of women in informed purchasing decisions.

Taken together, these findings confirm that PDO and PGI certifications are strategic instruments that not only communicate quality, but also contribute to territorial development, sustainability and cultural cohesion, provided that their attributes are reinforced through effective communication adapted to the different target audiences.

## 5. Conclusions and Limitations

This study confirms that Protected Designations of Origin (PDOs) and Protected Geographical Indications (PGIs) are not merely commercial labels but rather constitute strategic pillars within the agri-food sector and rural development. These certifications represent a comprehensive system that integrates quality, origin, tradition, sustainability, authenticity, and territorial identity. Over the past two decades, their influence has transcended the economic dimension, extending into the social, cultural, and environmental spheres, thereby establishing themselves as essential instruments for revitalizing rural areas.

In this regard, it can be asserted that quality certification schemes are not perceived by consumers as simple commercial marks; instead, they are regarded as guarantees of authenticity, excellence, and territorial linkage. This perception significantly contributes to the valorization of local products and enhances their competitiveness in the market.

It has been confirmed that product quality and geographical origin are the main attributes valued by consumers when making purchasing decisions. Although knowledge or greater knowledge about the designation of origin does not lead to greater acceptance of the products affected by a PDO and PGI, on the other hand, the respondents themselves are willing to pay more for these products. This connection between product and territory reinforces the cultural identity of producing regions and supports differentiation in competitive markets. However, although PDO and PGI certifications are often associated with more sustainable practices, environmental considerations have yet to emerge as decisive factors in consumer behavior. This presents an opportunity to improve communication regarding the ecological and social benefits associated with these certifications.

A clear generational gap has been identified in the knowledge of quality certification schemes. While older consumers exhibit higher levels of familiarity and appreciation for these certifications, younger audiences show significantly lower levels of awareness. This finding highlights the need to develop more modern communication strategies that incorporate digital tools and narratives tailored to younger generations.

From a quantitative perspective, the econometric analysis conducted in this study has demonstrated a relationship between the consumption of certified products, consumer awareness of the certification, and willingness to pay a premium. Specifically, a greater understanding of these certifications is associated with minor consumption levels and a clear willingness to pay more for certified products. This positive willingness to pay underscores the perceived added value attributed to certified products by consumers.

From a territorial standpoint, gastronomic tourism emerges as a key lever for rural development. A significant proportion of respondents reported having visited rural municipalities motivated by the presence of PDO or PGI products, recognizing their contribution to local employment, support for small producers, and efforts to counter depopulation. Certified products not only stimulate the local economy and create jobs but also attract visitors interested in authentic experiences rooted in regional food culture. In this way, certifications act as integrative mechanisms linking agri-food production, cultural identity, and tourism.

Finally, this study underscores the importance of embracing agri-food marketing as a critical tool for enhancing the visibility and perceived value of certified products. Implementing strategies that utilize digital media and communication focused on values such as sustainability, tradition, and excellence can significantly expand the consumer base—particularly among younger demographics—and strengthen the overall market position of PDO and PGI products both locally and globally.

In conclusion, PDO and PGI certifications should be understood as multifunctional instruments capable of generating economic value, promoting social cohesion, fostering environmental sustainability, and reinforcing cultural identity. To fully realize this potential, an integrated strategy is required—one that combines emotional and educational communication, cooperative development, the promotion of sustainable tourism, digital transformation, and the integration of sustainability throughout the agri-food value chain. Only through such a comprehensive approach can PDO and PGI schemes be consolidated as true engines of transformation in rural areas, ensuring their resilience, economic viability, and contribution to a more just, inclusive, and sustainable development model.

The findings suggest a need for the agri-food industry to promote greater awareness of PDO and PGI certifications among the general population. Moreover, expanding the number of certified products may be advisable, as consumer perception of these labels fosters a heightened sense of trust and security in their purchasing decisions.

At this point, we must acknowledge certain limitations. First, regarding the non-probabilistic sampling, for convenience, the sample was not entirely randomly selected, and instead depended on the accessibility and willingness of individuals to participate. This limits its representativeness of the general population. Individuals with a greater interest in the subject, a higher level of education, or more access to digital technologies may have been overrepresented.

During the completion of the open online questionnaire, it was not guaranteed that all participants belonged exclusively to the geographical area of interest (Extremadura), although filter questions were included to help characterize them. Regarding the self-administered nature of the questionnaire, the lack of direct supervision during its completion may have affected the quality of some responses or led to misinterpretations of the questions.

Due to the exploratory nature of this study and logistical limitations, we opted for non-probabilistic sampling for convenience in this work; we propose that in future studies, researchers expand the sample scope with probabilistic methods.

## Figures and Tables

**Table 1:** Key differences between PDO, PGI, and TSG.

Label	Full Name	Main Requirement	Link to Geography
PDO	Protected Designation of Origin	All production stages must occur in the defined area; quality is exclusive to the region	Strong
PGI	Protected Geographical Indication	At least one production stage must occur in the region; product has a reputation linked to its origin	Moderate
TSG	Traditional Specialty Guaranteed	Highlights traditional production methods or composition; no geographic link required	None or optional

**Table 2:** Cronbach’s alpha test.

Consumption Cp; Knowledge of Certification; Willingness to Pay for Certified Products; Sustainable Production; Rural Development; Age; Education Level	
Test scale = mean (unstandardized items)	
Reversed items: age, sex	
Average interitem covariance	383.721
Number of items in the scale	8
Scale reliability coefficient	0.86

Source: Authors’ own elaboration.

**Table 4:** Descriptive variables.

Variable	Obs	Mean		Min	Max
Knowledge	306	1.245	0.431	1	2
Consumption	306	25.023	42.221	1	99
Willingness to pay	306	25.121	42.167	1	99
Sustainability	306	25.065	42.192	1	99
Rural development	306	26.320	41.493	1	99
Age	306	3.899	1.446	1	6
Gender	306	1.327	0.470	1	2
Education level	306	3.438	1.121	1	5

Source: Authors’ own elaboration.

**Table 5:** Regression results.

Source	ss	df	MS	Nunber of Obs	=	306
				F(7, 298)	=	2579.56
Model	57.1612216	7	8.16588879	Prob > F	=	0
Residual	0.943	298	0.003	R-squared	=	0.9838
				Adj R-squared	=	0.9834
Total	58.1045752	305	0.190506804	Root MSE	=	0.056
**Consumption**	**Coef.**	**Std. Err.**	**t**	***p*** ** > I tI**	**[95% Conf. Interval]**	
**Knowledge**	−0.998	0.022	−44.01	0.000	−1.042	−0.953
**Willingness to pay**	0.036	0.011	3.220	0.001	0.139	0.058
**Sustainability**	−0.016	0.016	−1.020	0.308	−0.048	0.015
**Rural development**	−0.005	0.003	−1.650	0.099	−0.012	0.001
**Age**	−0.001	0.002	−0.440	0.659	−0.006	0.004
**Gender**	−0.005	0.007	−0.760	0.449	−0.019	0.008
**Education level**	0.005	0.003	1.640	0.102	−0.001	0.011
**Constant**	1.989	0.051	39.92	0.000	1.890	2.087

Source: Authors’ own elaboration.

## Data Availability

The original contributions presented in this study are included in the article. Further inquiries can be directed to the corresponding author.
